# Therapeutic Potential Target of Adenosine for Epilepsy: Focusing on Its Interaction with the Molecular Epileptogenic Network

**DOI:** 10.3390/biom16030453

**Published:** 2026-03-17

**Authors:** Xiaoning Zhao, Jiahui Deng, Zhonghua Xiong, Tianfu Li

**Affiliations:** 1 Department of Neurosurgery, Sanbo Brain Hospital, Capital Medical University, Beijing 100018, China; zhaoxiaoning@mail.ccmu.edu.cn (X.Z.); xiongzh123@mail.ccmu.edu.cn (Z.X.); 2 Department of Neurology, Sanbo Brain Hospital, Capital Medical University, Beijing 100018, China; 3 Beijing Key Laboratory of Epilepsy, Sanbo Brain Hospital, Capital Medical University, Beijing 100018, China; jiahui_deng@mail.ccmu.edu.cn; 4 Beijing Institute for Brain Disorders, Capital Medical University, Beijing 100018, China; 5 Laboratory for Clinical Medicine, Capital Medical University, Beijing 100018, China

**Keywords:** epilepsy, adenosine, adenosine receptor, adenosine kinase, epileptogenesis

## Abstract

Epilepsy is a neurological disorder characterized by a long-lasting predisposition to recurrently generate unprovoked seizures. Epilepsy affects over 70 million people worldwide, with approximately one-third suffering from pharmacoresistant seizures. Currently, the clinical antiseizure drugs lack efficacy in preventing epileptogenesis. Adenosine, as an endogenous anticonvulsant, inhibits the development of epilepsy via interaction with the molecular epileptogenic network on several levels: (i) Activation of A1 receptor inhibits glutamate release via presynaptic inhibition, and hyperpolarizes the synaptic potentials in postsynaptic neurons. (ii) The A2A receptor on astrocytes interacts with astroglial glutamate transporter GLT-1, controlling glial glutamate homeostasis. (iii) Activation of the A3 receptor inhibits GABA transporter type 1-mediated GABA uptake. (iv) Adenosine kinase (ADK) is highlighted as a pathological hallmark of epilepsy, with its distinct isoforms driving different mechanisms. The cytoplasmic short isoform (ADK-S) in astrocytes controls extracellular adenosine and receptor-mediated pathways, whereas the nuclear long isoform (ADK-L) in astrocytes and specific neurons regulates epigenetic mechanisms without relying on adenosine receptors. Collectively, this review clarifies the adenosine system’s critical regulatory role in the epileptogenic network, highlights adenosine receptors and ADK isoforms as promising therapeutic targets for epilepsy, and provides a theoretical basis for developing novel disease-modifying therapies for pharmacoresistant epilepsy while laying a foundation for subsequent preclinical and clinical translation.

## 1. Introduction

Epilepsy is a chronic neurological disorder characterized by recurrent abnormal synchronous neuronal discharges, affecting approximately 70 million people worldwide. The global prevalence of epilepsy varies with age, being higher in children and older age groups, with bimodal peaks at ages 5–9 years (374 per 100,000 population) and in those older than 80 years (545 per 100,000 population) [1]. The seizure types include focal (focal preserved consciousness seizure, focal impaired consciousness seizure, and focal-to-bilateral tonic–clonic seizure) versus generalized (absence seizure, generalized myoclonic seizure, generalized clonic seizure, generalized negative myoclonic seizure, generalized epileptic spasm, generalized tonic seizure, generalized atonic seizure, generalized myoclonic-atonic seizure, and generalized tonic–clonic seizure) [2]. Approximately 36% of these patients are insensitive to existing antiseizure medications (ASMs), leading to pharmacoresistant epilepsy [3,4]. Infantile Epileptic Spasms Syndrome (IESS), a developmental and epileptic encephalopathy with onset in infancy, presents with epileptic spasms and typically exhibits interictal hypsarrhythmia on electroencephalogram [5]. Dravet syndrome, a severe epileptic encephalopathy, is characterized by prolonged febrile hemiclonic seizures and developmental regression after age 1 year. Pathogenic variants in the sodium voltage-gated channel α subunit 1 (SCN1A) are identified in over 80% of cases [6]. Lennox–Gastaut syndrome, a severe epileptic encephalopathy, features varied origins, frequent seizure episodes like drop attacks, progressive mental impairment, and strong resistance to ASMs. The peak age of onset for epilepsy is 3–5 years [7]. Epileptic seizures in pharmacoresistant epilepsy cause brain neuronal damage [8,9,10], resulting in intellectual and psychiatric disorders, severely impairing patients’ quality of life [11]. Currently, ASMs primarily target neurons, mainly by modulating voltage-gated ion channels, augmenting gamma-aminobutyric acid (GABA)-ergic inhibition, or dampening excitatory neurotransmitter glutamate. Lacosamide selectively enhances the slow inactivation process of voltage-gated sodium channels, thereby inhibiting high-frequency neuronal firing and stabilizing overexcited neural networks. Levetiracetam specifically binds to the synaptic vesicle glycoprotein SV2A to regulate synaptic vesicle exocytosis and reduce the probability of excitatory neurotransmitter (e.g., glutamate) release, thereby inhibiting the excessive synchronization of epileptic networks. Brivaracetam binds to the synaptic vesicle glycoprotein SV2A with higher affinity and is more stable compared with Levetiracetam. In addition, it may also have an additional sodium channel blocking effect. Valproate inhibits seizures by increasing the inhibitory neurotransmitter GABA and inhibiting voltage-gated sodium channels. Cannabidiol inhibits seizures via modulation of intracellular calcium and inhibition of adenosine cellular uptake. However, these ASMs fail to significantly decrease the percentage of patients with pharmacoresistant epilepsy or intervene in the disease progression process effectively (i.e., epileptogenesis or disease modification) [12,13,14], and often come with severe systemic adverse effects. The crucial role of astrocyte dysfunction in the initiation and maintenance of epilepsy has been highlighted in recent years. Dysfunction in astrocytes likely contributes to ictogenesis and epileptogenesis via glial transmission dysfunction, cellular metabolic disruption (adenosine metabolism, glutamate and GABA metabolism), and immune and inflammatory dysfunction [14]. Metabolic pathways offer novel directions for drug development [15]. Particularly, the imbalance of glia-derived adenosine homeostasis is regarded as one of the key factors leading to neuronal hyperexcitability [14,16,17].

Adenosine is regarded as an endogenous anticonvulsant and neuroprotective regulator of the brain. Adenosine is maintained by the enzymes adenosine kinase (ADK) and adenosine deaminase (ADA), in conjunction with ectonucleotidases (CD39, CD73) and equilibrative nucleoside transporters (ENTs). Adenosine homeostasis in the brain is primarily governed by metabolic clearance through ADK levels under physiological conditions due to the high affinity of ADK for adenosine [18]. Increasing evidence has demonstrated that ADK is a marker of epileptogenesis and a target for its prevention [18,19,20,21,22,23,24,25]. There are two alternatively spliced forms of ADK: ADK-long (ADK-L), which is located in the nucleus, and ADK-short (ADK-S), which is located in the cytoplasm [26]. The cytoplasmic form of ADK-S regulates both intra- and extracellular adenosine levels; therefore, intracellular ADK-S controls the activation of adenosine receptors, thereby modulating neuronal excitability. In contrast, ADK-L is primarily located in the nucleus and is essential for sustaining methylation processes, such as DNA methylation [27,28,29], which are involved in controlling epileptogenesis [18].

Adenosine activates G protein-coupled receptors (GPCRs) with four subtypes: A1 receptors (A1Rs), A2A receptors (A2ARs), A2B receptors (A2BRs), and A3 receptors (A3Rs) [30]. Adenosine in the brain primarily regulates excitatory neurotransmission by activating G protein-coupled inhibitory A1Rs and excitatory A2ARs [30,31,32,33]. A disturbed balance between these receptors—such as reduced A1Rs signaling or enhanced A2ARs signaling—may promote the development and progression of epilepsy. A3Rs are regarded as a promising target for the development of antiseizure medications via A3R-mediated action upon GABA transporter type 1 (GAT-1) and GABAergic currents [34]. Currently, little is known about the contribution of A2BRs in epilepsy. Extensive evidence from experimental epilepsy models and research on human specimens demonstrates that aberrant adenosine signaling is a common pathologic feature in epilepsy [19,20,23,24,35,36]. Hence, targeting the adenosine system—via adenosine receptor agonists, adenosine kinase inhibitors, or gene therapy— holds therapeutic potential for epilepsy [21,37,38,39,40,41,42].

The present review is intended not to offer a comprehensive overview of all potential mechanisms, but to focus on the role of adenosine receptors (A1Rs, A2ARs, A3Rs), adenosine kinase in epilepsy, and propose perspectives on potential therapeutic targets (Table 1).

This is a narrative review. To ensure the comprehensiveness and timeliness of the included literature, we systematically searched the PubMed database using the following key terms: epilepsy, adenosine, adenosine A1 receptor, adenosine A2A receptor, adenosine A3 receptor, adenosine kinase, epileptogenesis, and DNA methylation. Two independent researchers extracted data from eligible articles in a single-blind manner, and a third senior researcher arbitrated discrepancies. All extracted data were double-checked for verification, with strict eligibility criteria (involving research theme, type, language, and data integrity) adopted to screen articles. Finally, we selected high-quality studies and synthesized their core findings to clarify the therapeutic implications of the adenosine system in epilepsy.

## 2. A1 Receptors (A1Rs) in Epilepsy

A1Rs belong to the GPCR family and mediate signal transduction through Gi/o proteins, leading to inhibition of cyclic adenosine monophosphate synthesis [43]. A1Rs are enriched in the central nervous system, where they are expressed in the cerebral cortex, hippocampus, cerebellum, thalamus, and brainstem, present at both pre- and postsynaptic sites in neurons, and also found in astrocytes, microglia, and oligodendrocytes. Adenosine has been proven to be a major endogenous anticonvulsant acting via A1Rs. In the brain, adenosine modulates neuronal activity via the following: (i) decreasing the presynaptic release of various neurotransmitters, and the most dramatic inhibitory actions are on the glutamatergic system through presynaptic A1Rs [44], and (ii) activating potassium ion channels, leading to hyperpolarization of postsynaptic neurons and promoting NMDA receptor inhibition through postsynaptic A1Rs [45]. Genetic deletion of A1Rs induces spontaneous electrographic seizures [24,36,40] and leads to lethal status epilepticus in mice models of epilepsy [60]. Downregulation of A1Rs in human temporal lobe epilepsy may contribute to the human epileptic condition [19,61]. Pharmacological blocking of adenosine A1Rs could transiently provoke seizures in epileptic animals treated with focal adenosine augmentation [42]. Deep-brain stimulation (DBS) markedly attenuated spontaneous recurrent seizures (SRSs) via an increase in extracellular adenosine [37], while pharmacological block of adenosine A1Rs reversed the effect of DBS on interictal epileptic discharges [21]. Pharmacological activation of A1R has been proven to mediate a strong anticonvulsant action in human neocortical slices from patients with temporal lobe epilepsy [62], as well as prevent the spread of seizures in mouse epileptic models. Of importance, in patients after a severe traumatic brain injury (TBI), variants in the A1R gene were associated with the development of post-traumatic seizures, which indicated that a deficiency in A1R signaling might be associated with post-traumatic epileptogenesis [63]. The development of A1R-targeting drugs faces challenges such as cardiovascular side effects (bradycardia, atrioventricular block, and diminished atrial contractility), sedation, as well as insufficient receptor selectivity [46].

## 3. A2A Receptors (A2ARs) in Epilepsy

A2ARs are coupled with stimulatory Gs proteins, facilitating cAMP production [43]. A2ARs are predominantly localized in the striatum, olfactory tubercle, and to a lesser extent in the cortex and hippocampus, with expression in neurons, astrocytes, microglia, and oligodendrocytes. A2ARs are pivotal in synaptic plasticity, counteract the A1Rs-mediated inhibition of synaptic transmission, increase expression in neurons and glial cells upon injury induced by seizures [20,35], and act as a STOP signal of the immune–inflammatory system [47]. By activating inhibitory A1Rs, adenosine raises the seizure threshold and promotes seizure termination, whereas stimulatory A2ARs may enhance synaptic transmission in a globally suppressed network. Therefore, alterations in crosstalk between A1R and A2AR signaling might be linked to seizure generation. Notably, sustained activation of high-affinity A1Rs can result in their internalization and a subsequent rise in A2AR expression [48]. Moreover, a specialized synaptic adenosine pool is governed by CD73, an enzyme that produces adenosine and thereby modulates A2AR activation at the synaptic level. The adenosine formed extracellularly via CD73 is a major driver of A2AR signaling. In line with this, increased immunoreactivity for both CD73 and A2ARs has been documented in brain specimens from patients with epilepsy associated with Rasmussen encephalitis [40], focal cortical dysplasia [20], and temporal lobe epilepsy [49].

Astroglial glutamate transporter-1 (GLT-1), the transporter responsible for the majority of glutamate clearance in the central nervous system, is essential for preventing seizures [50]. GLT-1 is under the control of A2ARs, which regulate astrocytic glutamate clearance through their physical associations with GLT-1 [51]. Increased A2AR expression coupled with diminished GLT-1 density in reactive astrocytes within the epileptic focus in patients with Rasmussen encephalitis [35] and focal cortical dysplasia [20] further supported A2AR-mediated modulation of glutamate uptake. Currently, most research using both selective antagonists and genetic manipulations of A2ARs has consistently substantiated proconvulsant effects of endogenously activated A2ARs. Both selective antagonists and genetic ablation of A2ARs markedly reduced both the incidence and severity of seizures, supporting a proconvulsant role of A2ARs via modulation of excitatory neurotransmission [52,53]. A2AR expression was found to be enhanced in epileptic WAG/Rij rats, a genetic model of human absence epilepsy [54]. Of importance, the dual modulation of A2ARs and GLT-1 represents a synergistic approach to attenuate brain hyperexcitability, with therapeutic potential for mitigating TBI-related persistent hyperexcitability. Therefore, targeting A2ARs and glial glutamate transporter GLT-1 synergistically might be a novel disease-modifying approach [57]. A2ARs, the primary modulators of excitatory transmission and neuronal excitability, exhibit increased expression in epilepsy. However, the causality of these changes—whether they drive epilepsy or are secondary adaptations—remains unresolved [64].

Activation of A2ARs has mainly proconvulsive actions. However, the literature contains important contradictions. A2ARs indicate both proconvulsant and anticonvulsant roles depending on brain region, seizure model, and timing. In the acute seizures model induced by kainic acid, the adenosine A2AR agonist CGS21680 provides protection against excitotoxicity [55]. CGS21680 contributes to the suppression of seizures in audiogenic-seizure-sensitive DBA/2 mice model, a commonly used generalized reflex epilepsy model [56]. In a model of temporal lobe epilepsy induced by pilocarpine in rats, A2AR antagonist DMPX shortened the latency of status epilepticus, demonstrating a proconvulsant effect [65]. In mice with striatal and extrastriatal A2AR knockouts, contrary to the role of intrastriatal A2ARs, A2ARs of extrastriatal neurons significantly promote the effects of psychostimulants, and A2AR agonist CGS21680 paradoxically attenuates seizure activity [66]. The proconvulsant effect of the A2AR antagonist DMPX might be related to the A2A/A1 selectivity ratio of DMPX [67], and the anticonvulsant effect of A2AR agonist CGS21680 might be associated with the low affinity of CGS21680 for adenosine A1Rs [68]. Therefore, more precise design studies are needed to address the role of A2ARs in epilepsy.

## 4. A3 Receptors (A3Rs) in Epilepsy

Similarly to A1Rs, A3Rs belong to the GPCRs family and mediate signal transduction through Gi/o proteins, leading to inhibition of cyclic adenosine monophosphate synthesis. A3Rs are widely expressed at hippocampal synapses, both pre- and postsynaptically, with expression in neurons [69], astrocytes [70], and microglia [71]. The role of the A3Rs in epilepsy, through the regulation of the GAT-1-mediated GABA transport mechanism, provides new targets and strategies for modulating the balance of central inhibitory neurotransmitters. Its activation inhibits GAT-1 function, increases GABA concentration in the synaptic cleft, thereby enhancing inhibitory signals, reducing neuronal excitability, and alleviating the frequency and intensity of epileptic seizures [34]. Compared to A1Rs, A3Rs might be a more ideal target for epilepsy therapy, such as: (i) without side effects on cardiac function, as a non-ubiquitous adenosine receptor, A3Rs are a putative target for novel antiseizure medications. Adenosine has been proven to be a major endogenous anticonvulsant acting via A1Rs. However, the widespread distribution of A1Rs throughout the body raises the concern that A1R agonists may lead to bradycardia. (ii) Minimal sedative effects: the A3R antagonist has selective actions in epileptic tissue (rodent and human), sparing non-epileptic tissue [58]. A3R antagonists selectively affect epileptic tissue while preserving excitatory transmission in healthy tissue [34]. (iii) The upregulation of A3Rs versus downregulation of A1Rs in epileptic focus: A decrease in A1Rs [19,59] while an increase in A3Rs [34] in epileptic tissue has been demonstrated in patients and the majority of studies in animal models, which indicates that activation of A3Rs might contribute to more antiseizure potential. A3Rs agonists, as a potentially safe new class of antiseizure medications, are still in the early stages of development from bench to bed for epilepsy, with key issues including blood–brain barrier permeability, long-term safety, and efficacy stability requiring further attention [34].

## 5. ADK-S in Epilepsy: Modulating Extracellular/Synaptic Adenosine Levels and Inhibiting Seizures via Adenosine Receptor-Dependent Way

**Figure 1:**
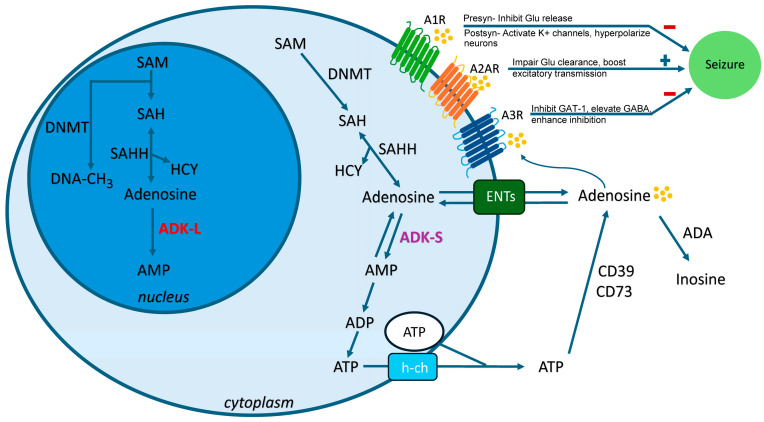
The role of adenosine in epilepsy: subcellular regulation and mechanisms in epilepsy. Adenosine is localized in extracellular, cytoplasmic, and nuclear compartments. (i) Extracellular adenosine is maintained by ATP degradation via ectonucleotidases (CD39/CD73), ADA catabolism, and equilibrative nucleoside transporters (ENTs). Extracellular adenosine mainly activates adenosine receptors (ARs: A1Rs/A2ARs/A3Rs), modulating neuronal excitability. (ii) Cytoplasmic adenosine arises from transmethylation reactions and is regulated by ADK-S, with metabolism by ADA and ENTs playing key roles; ATP is released from astrocytes via hemichannels (h-ch), a critical source of extracellular adenosine. Cytoplasmic adenosine regulates transmethylation by DNMT (DNA methylation), influencing neurotransmitter balance. (iii) Nuclear adenosine, generated by transmethylation, is controlled by ADK-L. Nuclear adenosine controls epigenetic mechanisms via DNMT, potentially affecting gene expression in epilepsy. This compartmentalized regulation highlights adenosine’s dual role in both suppressing seizures (via A1Rs activation) and contributing to neuroplasticity (via epigenetic modulation).

On the one hand, ADK-S modulates extracellular/synaptic adenosine. ADK-S is primarily located in the cytoplasm, regulating extracellular/synaptic adenosine levels, thereby modulating the activation of adenosine receptors [72]. The primary source of synaptic adenosine is ATP, which is released through vesicular transport [73] or via hemichannels in astrocytes [74]. After entering the synaptic cleft, ATP is degraded into adenosine by a series of extracellular nucleotidases (CD39 and CD73) [75]. Astrocytic membranes contain equilibrative nucleoside transporters (ENTs), which rapidly balance extracellular and cytoplasmic adenosine levels [76]. Intracellular adenosine levels are largely controlled by ADK-S, which phosphorylates adenosine into AMP. Due to the lack of a classical transporter-regulated reuptake system for adenosine and its high affinity for adenosine, cytoplasmic astrocytic ADK-S is considered to be a metabolic reuptake system for adenosine, causing an inward net flux through ENTs and maintaining a low extracellular adenosine level [18]. Extracellular adenosine plays an important role in epilepsy by modulating neuronal excitability via G protein-coupled adenosine receptors. On the other hand, ADK-S modulates cytoplasmic adenosine concentrations and influences the transmethylation process. The transmethylation pathway provides an additional source of intracellular adenosine beyond ATP breakdown [77]. DNA methylation is catalyzed by DNA methyltransferases (DNMTs), which transfer a methyl group from S-adenosylmethionine (SAM) to the fifth carbon atom of cytosine, forming 5-methylcytosine (5-mC). Concurrently, SAM is converted to S-adenosylhomocysteine (SAH), which is then hydrolyzed by SAH hydrolase (SAHH) to produce adenosine and homocysteine (HCY) (Figure 1). Moreover, the adenosine produced through the transmethylation pathway is primarily recycled by the cytoplasmic isoform ADK-S [78]. Of note, the thermodynamic equilibrium of the SAH hydrolysis reaction favors SAH formation. As a result, this key reaction—responsible for modulating methyl group flux throughout the transmethylation pathway—requires low intracellular adenosine concentrations [79] and efficient adenosine clearance by ADK [80]. DNA methylation is one of the most important types of epigenetic modification. Studies on brain tissue from patients with temporal lobe epilepsy (TLE) and TLE animal models suggest that DNA methylation is involved in the pathophysiological process of TLE and plays a critical role in the pathogenesis of epilepsy [39,81,82,83,84]. Nevertheless, the potential regulation of cytosolic methyltransferase activity by adenosine and ADK levels has not yet been fully elucidated and requires further exploration [28].

Adenosine is an endogenous neuromodulator released during seizures and implicated in seizure arrest, postictal refractoriness, and suppression of epileptogenesis [85]. Therefore, ADK-S inhibition is a potential strategy to boost extracellular adenosine for epilepsy control. This concept is supported by multiple lines of evidence: (i) Animal Models: ADK-overexpressing transgenic mice show increased sensitivity to brain injury and seizures, whereas mice with reduced forebrain ADK are resistant to pharmacologically induced epileptogenesis [36]. (ii) Viral Induction: Wild-type mice with virus-induced ADK overexpression in hippocampal CA3 astrocytes develop electrographic seizures [86]. (iii) Genetic Intervention: Intrahippocampal implantation of stem cells with biallelic ADK gene disruption (Adk^−/−^) prevents kainate-induced epileptogenesis in a limbic mouse model [36]. (iv) Human Studies: ADK overexpression is observed in human temporal lobe epilepsy tissue [19], focal cortical dysplasia tissue [20], Rasmussen encephalitis tissue [23], Sturge–Weber Syndrome tissue [87] and glial tumor tissue as well as peritumoral regions infiltrated by glia [88], suggesting that reduced adenosine may contribute to epileptogenesis in these conditions. Additionally, basal adenosine levels are lower in the epileptic human hippocampus compared to controls [85], further supporting ADK’s potential role in epileptogenesis. (v) Dietary Therapy: A ketogenic diet suppresses seizures in ADK-overexpressing transgenic mice and A1R^+/−^ mice by inhibiting ADK expression, thereby increasing ambient adenosine and activating A1Rs [40]. The suppression of seizures induced by the diet was reversed by either glucose administration (metabolic reversal) or an A1R antagonist (pharmacological reversal). Notably, in A1Rs-deficient mice, the diet failed to influence spontaneous seizures. Combined pharmacological and genetic evidence thus strongly indicates that the diet’s anticonvulsant action is exclusively mediated by A1R activation, aligning with the proposed therapeutic role of adenosine signaling in epilepsy control. (vi) Neuromodulation Therapy: DBS of the anterior nucleus of the thalamus (ANT) can reduce the frequency of spontaneous recurrent seizures (SRS) and attenuate seizure progression in epileptic rats via inhibition of ADK [21,89]. DBS significantly elevated hippocampal adenosine levels via in vivo measurements of adenosine using fiber photometry [37] or microdialysis [90], which might be derived from the downregulation of ADK. A1Rs antagonists reversed the effect of DBS on interictal epileptic discharges in epileptic rats [21]. In vitro experiments demonstrated that the DBS-induced reduction in neuronal excitability was completely blocked in animals pretreated with A1R antagonists, whereas it was markedly enhanced by A1R agonists. The findings indicate that DBS can reduce SRS in epileptic rats via inhibition of ADK-S, the subsequent increase in extracellular/synaptic adenosine levels, and activation of A1Rs, which are consistent with the proposed role of adenosine signaling in epilepsy control. Therefore, ADK-S plays a critical role in maintaining adenosine receptor-mediated signal transduction, and changes in its activity directly affect the availability of extracellular/synaptic adenosine and the extent of adenosine receptor activation. The characteristic that inhibition of ADK-S can effectively elevate extracellular/synaptic adenosine levels makes it a potential therapeutic target for seizure suppression.

## 6. ADK-L in Epilepsy: Modulating Adenosine Receptor-Independent Epigenetic Mechanisms

Distinct from its cytoplasmic ADK-S counterpart, ADK-L is specifically localized in the nucleus [26]. Within the nucleus, ADK-L regulates adenosine metabolism to control DNA methylation through transmethylation reactions driven by DNA methyltransferases (DNMTs) (Figure 1). The dynamic regulation of DNA methylation is directly mediated by the adenosine-responsive transmethylation pathway. Hippocampal infusion of adenosine, the end product in the transmethylation pathway, induces global DNA hypomethylation, while infusion of SAM, the primary methyl donor for transmethylation reactions, causes hypermethylation indirectly [39]. Pharmacological ADK inhibition (5-iodotubercidin, 5-ITU) or genetic ADK reduction leads to brain DNA hypomethylation. Notably, 5-ITU-dependent hypomethylation persisted in mice lacking the adenosine A1Rs, demonstrating that receptor activation—the primary mechanism underlying the anticonvulsant effects of adenosine [33]—is dispensable for triggering ADO-induced hypomethylation. Of importance, increased levels of both ADK-L and ADK-S isoforms contribute to elevated global DNA methylation (400% versus 50%), with the nuclear isoform ADK-L playing a more dominant role in regulating DNA methylation status [39]. These findings establish ADK-L as an epigenetic regulator independent of adenosine receptor signaling. On the one hand, extensive evidence suggests that upregulation of DNA methylating enzymes and DNA hypermethylation play a role in the pathogenesis of human and experimental epilepsy, especially as DNA methylation makes a contribution to epileptogenesis [81,83,84,89,91,92,93]. On the other hand, ADK-L plays a chief role in regulating DNA methylation status [39,94], and the disease-modifying effects of adenosine in epilepsy may be mediated by its regulation of epigenetic processes. Therefore, blockade of ADK-L may contribute to modifying the disease course of epilepsy or prevent epileptogenesis through an epigenetic mechanism. Recently, novel ADK-L inhibitors developed through structure-based approaches (MRS4380/MRS4203) offer enhanced potency over traditional nucleoside-based inhibitors [95]. These compounds demonstrate concentration-dependent DNA hypomethylation effects in cancer cells [94], highlighting their potential as epigenetic modulators. Future development requires optimizing nuclear entry, ADK-L selectivity, and the ability to modulate DNA methylation, as well as achieving sustained antiepileptic effects through refined structure-based design.

Clinical therapies for pharmacoresistant epilepsy—such as ketogenic diet, DBS, and vagus nerve stimulation (VNS)—highlight the clinical utility of ADK-L modulation for therapeutic benefits as follows: i) Dietary Therapy: Epilepsy rodent models reveal persistent disease-modifying effects of ketogenic diet therapy, driven by epigenetic mechanisms [38,84,96]. Likewise, a transient use of ketogenic diet therapy in a subset of children with epilepsy can yield lasting seizure freedom after discontinuation of the diet [97,98]. Furthermore, ketogenic diet therapy increases adenosine levels [40,96] while decreasing adenosine kinase expression [40]. These findings suggest that the sustained therapeutic benefits of ketogenic diet therapy after discontinuation may be attributed to epigenetic changes triggered by elevated adenosine and ADK-L modulation. ii) DBS therapy: The kindling process of the epilepsy model in DBS therapy is as follows: The rats were intraperitoneally injected with pentylenetetrazol (PTZ) three times a week for 6 weeks (a total of 18 injections). When a rat exhibited generalized seizures after three consecutive injections, it was considered to be “fully kindled.” After the eighteenth kindling stimulus, the rats were housed for 7 days (without PTZ or DBS) and then received a single challenge dose of PTZ. DBS disrupts the kindling process, with seizure suppression persisting even one week after stimulation cessation. These results indicate that ANT-DBS may offer therapeutic advantages in slowing epilepsy progression, as well as disease-modifying benefits, or potential antiepileptogenic effects [89]. In addition, DBS therapy increases adenosine levels [37,90] while decreasing adenosine kinase expression [40]. Elevated ADK-L expression during epileptogenesis promotes DNA hypermethylation, a mechanism independently demonstrated to contribute to epileptogenesis. These results demonstrate that the therapeutic benefits of DBS for disease modification are due to elevated adenosine and ADK-L modulation. iii) VNS therapy: For patients with drug-resistant epilepsy ineligible for resective surgery, VNS offers a viable treatment option. The therapy reduces seizure frequency by more than half in 50–60% of recipients, and 6–11% become entirely seizure-free [99]. Optimized stimulation parameters (higher duty cycle and output current) were associated with better seizure control and substantial subjective improvements in quality of life [100]. The kindling process of the epilepsy model in VNS therapy is as follows: Once the threshold of electrical stimulation was reached in cats, amygdala kindling was performed every 24 h with amygdala stimulation. The vagus nerve was previously stimulated for 1 min just before amygdala kindling and every 60 min thereafter, four times a day. Compared with the control group (behavioral stage VI), amygdala kindling was significantly delayed in the VNS group, with animals remaining in behavioral stages I–III. The results indicate that VNS has antiepileptogenic properties [101]. Evidence from diverse studies suggests that DBS activates adenosine release [37,90,102]. Genetic variations in the Adk gene have been identified as a reliable biomarker for predicting the effectiveness of VNS treatment. All patients with homozygosity for the minor allele in specific Adk single-nucleotide polymorphisms (SNPs), specifically homozygous rs11001109 (AA) and rs946185 (AA), achieved > 50% seizure reduction, and 40% of these patients attained complete seizure freedom [22]. In addition, VNS inhibits DNA methylation in a rat model of pilocarpine-induced temporal lobe epilepsy [81]. These discoveries underscore a pivotal role of ADK-L in VNS therapy and reinforce the idea that the enzyme plays an intricate role in the pathogenesis of epilepsy.

## 7. Future Perspective

A3R agonists represent a promising novel class of antiseizure medications with an improved safety profile, currently undergoing early-stage development from preclinical research to clinical application for epilepsy treatment. However, their therapeutic potential hinges on addressing critical challenges, including optimizing blood–brain barrier permeability to ensure sufficient central nervous system exposure, establishing long-term safety profiles through comprehensive preclinical and clinical evaluation, and ensuring sustained efficacy stability during chronic administration.

Novel inhibitors with preferential inhibition of ADK-L over ADK-S are particularly promising. The enduring disease-modifying effects of adenosine are rooted in an epigenetic and receptor-independent mechanism regulated by ADK-L, which orchestrates DNA methylation to sustain long-term therapeutic outcomes and avoids the cardiovascular and sedative side effects of adenosine, which arise from excessive activation of adenosine receptors (linked to ADK-S).

For ASMs and clinical trials, precision therapy and synergistic combinations of repurposed drugs for antiepileptogenesis are promising in the near future. Recent studies have found that antisense oligonucleotide (ASO) gene therapy holds certain promise in treating Dravet syndrome. Dravet syndrome is caused by a loss-of-function mutation in SCN1A. In mouse models of Dravet syndrome, ASO treatment increases SCN1A gene expression, thereby reducing seizure frequency. It is reported that two Phase I/IIa clinical trials for ASO gene therapy have already begun [103]. In addition, synergistic combinations of repurposed drugs might prove to be effective in preventing epilepsy in patients at risk within the next decade [104].

While animal models are crucial for uncovering epilepsy mechanisms and testing therapies, their limitations must be acknowledged. Species differences, the complexity of human epilepsy, and the diversity of clinical phenotypes necessitate cautious extrapolation of results. Rigorous preclinical validation, large-scale long-term clinical trials, and verification using human-relevant models (such as organoids and stem cell models) are essential to ensure safe and effective translation of research.

## 8. Conclusions

The adenosine system, as a core component of the endogenous antiepileptic network, regulates neuronal excitability through various receptors and metabolic pathways. Through interacting with the molecular epileptogenic network on several levels, adenosine has been shown to inhibit the development of epilepsy. From molecular mechanisms to models and clinical translation, substantial evidence supports the critical value of adenosine-related targets in epilepsy treatment. Intervention strategies such as global ADK inhibitors, A1Rs agonists, and A2ARs antagonists demonstrate promising antiepileptic potential. However, overcoming peripheral side effects and achieving precise intervention through targeted delivery remain major challenges in clinical translation.

## Figures and Tables

**Table 1:** Adenosine receptor subtypes (A1Rs, A2ARs, A3Rs) in epilepsy: expression, mechanisms and therapeutic implications.

Receptor Subtypes	Expression Patterns	Mechanism of Action	Therapeutic Implications
A1Rs	- Tissue distribution: Widely expressed in CNS (cerebral cortex, hippocampus, cerebellum, thalamus, and brainstem). - Cellular localization: Present in pre- and postsynaptic neurons, as well as astrocytes, microglia, and oligodendrocytes.	- Signal transduction: Couples to Gi/o proteins, inhibiting cAMP synthesis [43]. - Neuronal regulation: 1. Presynaptic: Reduces glutamate release to suppress neuronal overexcitation [44]. 2. Postsynaptic: Activates potassium ion channels, hyperpolarizes neurons, and inhibits NMDA receptors [45].	- Therapy interaction: Mediates the antiepileptic effects of DBS and ketogenic diet (inhibiting ADK to increase adenosine, which activates A1Rs). - Potential interventions: Agonists exhibit strong anticonvulsant effects (e.g., suppressing seizure spread in mouse models and carbamazepine-resistant seizures in human neocortical slices). - Challenges: Peripheral side effects (bradycardia, atrioventricular block) and insufficient receptor selectivity limit clinical application [46].
A2ARs	- Tissue distribution: Predominantly localized in the striatum and olfactory tubercle; weakly expressed in the cortex and hippocampus. - Cellular localization: Expressed in neurons, astrocytes, microglia, and oligodendrocytes.	- Signal transduction: Couples to Gs proteins, promoting cAMP production [43]. - Dual roles: 1. Proconvulsant effects: Enhances excitatory neurotransmission by counteracting A1R-mediated inhibition; reduces astrocytic glutamate clearance via impaired interaction with astroglial glutamate transporter GLT-1 [47,48,49,50,51,52,53,54]. 2. Anticonvulsant effects: Protects against excitotoxicity in kainate-induced acute seizures; suppresses audiogenic seizures in DBA/2 mice [55,56].	- Potential interventions: 1. Selective antagonists: Reduces seizure incidence/severity in animal models (e.g., WAG/Rij rats with absence epilepsy) [53]. 2. Synergistic targeting: Dual modulation of A2ARs and GLT-1 alleviates traumatic brain injury (TBI)-related brain hyperexcitability [57]. - Challenges: Effects depend on brain region, seizure model, and timing; need precise design to avoid contradictory outcomes.
A3Rs	- Tissue distribution: Widely expressed at hippocampal synapses (pre- and postsynaptic sites). - Cellular localization: Expressed in neurons, astrocytes, and microglia.	- Signal transduction: Couples to Gi/o proteins, inhibiting cAMP synthesis. - Neuronal regulation: Inhibits GAT-1-mediated GABA uptake, increasing GABA concentration in the synaptic cleft to enhance inhibitory signals and reduce neuronal excitability [34].	- Potential interventions: Agonists are promising novel antiseizure candidates with advantages: 1. Safety: No cardiac side effects (unlike A1Rs) and minimal sedation (selective for epileptic tissue) [58]. 2. Efficacy: Targets the upregulated A3Rs in epileptic foci, with stronger antiseizure potential than A1Rs [34,59]. - Challenges: Early development stage; needs optimization of blood–brain barrier permeability, long-term safety, and efficacy stability [34].

## Data Availability

No new data were created or analyzed in this study. Data sharing is not applicable to this article.

## Abbreviations

The following abbreviations are used in this manuscript:

5-ITU	5-iodotubercidin
5-mC	5-methylcytosine
A1Rs	A1 receptors
A2ARs	A2A receptors
A2BRs	A2B receptors
A3Rs	A3 receptors
ADA	adenosine deaminase
ADK	adenosine kinase
ADK-L	long isoform of adenosine kinase
ADK-S	short isoform of adenosine kinase
ANT	anterior nucleus of the thalamus
ASMs	antiseizure medications
ASO	antisense oligonucleotide
DBS	deep brain stimulation
DNMTs	DNA methyltransferases
ENTs	equilibrative nucleoside transporters
GABA	gamma-aminobutyric acid
GAT-1	GABA transporter type 1
GLT-1	glutamate transporter-1
GPCR	G protein-coupled receptor
h-ch	hemichannel
HCY	homocysteine
IESS	Infantile Epileptic Spasms Syndrome
PTZ	pentylenetetrazol
SAH	S-adenosylhomocysteine
SAHH	SAH hydrolase
SAM	S-adenosylmethionine
SCN1A	sodium voltage-gated channel α subunit 1
SNPs	single nucleotide polymorphisms
SRSs	spontaneous recurrent seizures
TBI	traumatic brain injury
TLE	temporal lobe epilepsy
VNS	vagus nerve stimulation
